# Jean Schoenen, David W. Dodick, Peter S. Sándor: Comorbidity in Migraine: Wiley-Blackwell, 2011

**DOI:** 10.1007/s10194-011-0389-2

**Published:** 2011-10-01

**Authors:** Antonio Carolei

**Affiliations:** Department of Neurology, University of L’Aquila, 67100 L’Aquila, Italy

This excellent and concise book is dedicated to clinicians involved in the care of patients suffering from migraine. Migraine has been found to be comorbid with several disorders. The presence of a comorbid condition in a migraine patient may enhance the disability of migraine itself and in some cases comorbid conditions may be represented by disabling or life-threatening diseases. Proper knowledge of the spectrum of migraine comorbidities may offer hints to better understand the etiopathogenesis of migraine as to add numerous advantages to the management of the patients. The choice of the most appropriate migraine treatment must take into account comorbidities and vice versa. Some treatments have the potential to cure simultaneously migraine and the comorbid disorder thus improving compliance and prognosis while others should be avoided in the presence of the comorbid disorder. All clinicians involved in the management of migraine patients should be aware of those notions.

The book is structured into ten chapters, all written by well-known and qualified contributors, dealing with relevant aspects such as Psychiatric Comorbidity in Migraine; Migraine and Stroke; Cardiovascular Disorders; Patent Foramen Ovale and Migraine; Comorbidity of Migraine and Epilepsy; Migraine and Other Pain Disorders; Migraine and Medication Overuse; Migraine and other Comorbidities: Obesity, Temporomandibular Disorders, and Contact Points; Migraine Comorbidities in Children; and Optimal Management of Migraine Taking into Account Comorbidities and Positive Side Effects. All chapters go through the topic of interest thoroughly, giving insights on the mechanisms of the comorbidity, on clinical aspects, diagnosis, and management. The inclusion of Case Reports that may be of help both for the clinicians and the less expert readers adds to the general appreciation for the book itself as the very useful and up to date bibliography. I recommend this excellent and concise book not only to clinicians interested in migraine but also to medical practitioners, residents in neurology, cardiology, psychiatry, child psychiatry, internal medicine, anesthesiology, general practitioners, and medical students as an opportunity to get a better knowledge of the topic.

